# Self-Correcting Recurrent Neural Network for Acute Kidney Injury Prediction in Critical Care

**DOI:** 10.34133/2021/9808426

**Published:** 2021-12-23

**Authors:** Hao Du, Ziyuan Pan, Kee Yuan Ngiam, Fei Wang, Ping Shum, Mengling Feng

**Affiliations:** ^1^Saw Swee Hock School of Public Health, National University of Singapore, Singapore; ^2^School of Electrical and Electronic Engineering, Nanyang Technological University, Singapore; ^3^National University Health System, Singapore; ^4^Population Health Sciences, Weill Cornell Medical College, Cornell University, New York, USA

## Abstract

*Background*. In critical care, intensivists are required to continuously monitor high-dimensional vital signs and lab measurements to detect and diagnose acute patient conditions, which has always been a challenging task. Recently, deep learning models such as recurrent neural networks (RNNs) have demonstrated their strong potential on predicting such events. However, in real deployment, the patient data are continuously coming and there is no effective adaptation mechanism for RNN to incorporate those new data and become more accurate.*Methods*. In this study, we propose a novel self-correcting mechanism for RNN to fill in this gap. Our mechanism feeds prediction errors from the predictions of previous timestamps into the prediction of the current timestamp, so that the model can “learn” from previous predictions. We also proposed a regularization method that takes into account not only the model’s prediction errors on the labels but also its estimation errors on the input data.*Results*. We compared the performance of our proposed method with the conventional deep learning models on two real-world clinical datasets for the task of acute kidney injury (AKI) prediction and demonstrated that the proposed model achieved an area under ROC curve at 0.893 on the MIMIC-III dataset and 0.871 on the Philips eICU dataset.*Conclusions*. The proposed self-correcting RNNs demonstrated effectiveness in AKI prediction and have the potential to be applied to clinical applications.

## 1. Introduction

Electronic health record (EHR) data are accumulative, routinely collected patient observations from hospitals or clinical institutes. In the case of intensive care unit (ICU), EHR includes not only the static information such as patient demographics and discrete time series data such as medication and diagnosis but also continuous multivariate time series data such as vital signs and laboratory measurements. In order to detect and diagnose acute (and usually deadly) patients’ conditions, ICU intensivists need to continuously monitor high-dimensional vital signs and lab measurements [1]. Example acute conditions include acute kidney injury, acute hypertension, acute organ failure, and acute septic shocks. It has always been challenging to track all indicative changes in various patients’ data to diagnose these acute conditions accurately in time. Predictive models developed with ICU EHR data provide an opportunity for early detection of ICU acute conditions, which can lead to in-time and better care. In this study, we propose a self-correcting deep learning framework with accumulative ICU data to predict these acute conditions. Without the loss of generality, we will focus on the prediction of acute kidney injury (AKI), while the predictive modeling of other conditions can be handled similarly.

AKI is a sudden onset of renal failure or kidney damage, occurring in at least 5% of hospitalized patients. It is associated with a significant increase on mortality, length of stay (LOS), and hospital cost under a wide range of conditions [2]. AKI is a good study case for disease risk predictive modeling because (1) the precise definition for AKI allows temporal anchoring of events. AKI can be defined with the urine output criteria in a short diagnostic time frame. These criteria require dynamic modelling over a rolling 6 to 24 hours’ window [3]. The hourly recorded EHR data in ICU makes it possible to identify the onset of AKI events accurately and efficiently. (2) If detected and managed in time, AKI is potentially avoidable and reversible in the process of a few hours to several days. A previous trial showed that AKI alerts in the ICU led to an increase in the frequency of therapeutic interventions for AKI patients and that AKI patients in the “risk” phase were more likely to return to baseline renal function within 8 hours [4]. An accurate, automated, and early AKI detection system could potentially prevent AKI events, thus reducing mortality, shortening LOS, avoiding the development of chronic kidney disease, and creating quality of care indicators.

AKI prediction with utilization of features from EHR data is attracting widespread research interest. In particular, much research in recent years has focused on predictive modeling on a broad population to identify high-risk subjects as early as possible [5]. Initially, AKI prediction was modeled by standard statistical modeling methods, including logistic regression, discriminant analysis, or decision tree algorithm [6– 9]. Data were accumulated using sliding window method, and the prediction was generated at a specified interval (per hour, two hours, day, shift, etc.). Recently, a number of studies have been carried out utilizing recurrent neural networks (RNNs) in clinical diagnosis and prediction. RNNs are one branch of neural networks, which are powerful to process sequential data [10]. In RNNs, hidden units connect to each other by forming a directed cycle. Each output value is dependent on the previous computations. In traditional RNNs, the network can only look back to a few steps due to the gradient vanishing and exploding problems [10]. To address these limitations, variants of RNNs, such as LSTM [11] and GRU [12], are proposed and well utilized in clinical prediction problems. These variants have the hidden state with the forget gate that decide what to keep in and erase from the memory of the network.

In addition to LSTMs, pooling and word embedding were used in DeepCare [13] to model illness states of patients and to predict patients’ outcomes. DeepCare is an end-to-end deep dynamic memory neural network. DeepCare introduced time parameterizations to handle irregularly timed events and utilized accumulative temporal data by moderating the forgetting and consolidation of memory cells. DeepCare demonstrated improved disease progression modeling accuracy and risk prediction compared to Markov models and plain RNNs. The limitation that is shared by both Doctor AI and DeepCare is that, as they continue to predict patients’ disease progression, they lack a feedback mechanism to allow the models to learn and improve from their previous prediction mistakes.

Specifically, on AKI prediction, a number of studies have been carried out. Hurry et al. employed Bayesian networks on AKI prediction by predicting the likelihood of AKI onset based on longitudinal patient data on the MIMIC II database [14]. In addition, Nogueira et al. applied Markov chain model on the PhysioNet dataset to predict the future state of the patients based on the current medical state and ICU type. The common limitation of these studies is that the proposed methods are all dependent on hand-engineered features and expert knowledge, where the hidden states of the patient condition were not effectively modeled.

In the setting of ICU, or more in general the setting of in-patient care, after patients’ admission, their situation often evolves rapidly over time. Therefore, patients’ EHR data are dynamic time series in nature. The challenges of longitudinal EHR data, including event temporality, high dimensionality, and irregular sampling, have been investigated in many studies using RNNs [15]. In addition to these challenges, patients’ EHR data also have an accumulative characteristic: as patients stay longer in the hospital, more data are collected about their disease progression, and thus a more accurate modeling of patients’ physiological states is possible. Conventional RNNs were not optimized to accumulate information in time series data. The accumulated error between prediction and the patient’s status are not specifically modeled across the patients’ ICU stay. In addition, an effective ICU acute condition predictor is expected to enhance itself through self-correcting and learning from its accumulated prediction errors. This self-correcting mechanism is lacking in conventional RNN models.

To address the above limitations, we propose a variant of RNN to predict the onset of patients’ AKI in ICU. In this pilot study, we validate the effectiveness of our proposed self-correcting model with two actual ICU patient EHR datasets from the US. In the next phase, we plan to validate and deploy our algorithm in our local hospital.

The main contributions of this study are summarized as follows:

(1) Our method utilized the accumulative data of patients in ICU instead of a snapshot of the patient’s condition to improve the performance of AKI prediction
(2) We developed a novel accumulative self-correcting mechanism by modeling the accumulated errors in the model when the prediction is incorrect
(3) We proposed a regularization method for our model, which takes into account not only the model’s prediction error on the label but also its estimation errors on the future input data. Such regularization reduces the variance of the model and improves the efficiency of the self-correcting mechanism
(4) Our proposed method has been validated in two real-world large-scale ICU datasets. It was shown to outperform traditional RNNs. In addition, the method is in-progress of being validated locally with data from our own hospital

## 2. Materials and Methods

### 2.1. Problem Definition

For any ICU patient and any time point t during his/her ICU stay, our goal is to predict whether the ICU patient may develop AKI in next 6 hours, i.e., t+6, based on all his/her data of this ICU stay accumulated up till time t . In this study, AKI was defined according to the most commonly used RIFLE criteria [16]. A patient was detected with AKI if his/her urine output is less than 0.5 mL/kg/h for ≥6 h. 

Based on this definition, for a patient, the AKI actual onset label at any time t, denoted as $y_{t}$, can only be observed at time step t+6. In the traditional RNN, the correctness of the predicted ${\hat{y}}_{t}$ would not affect the prediction in the future time step; although at time step t+6, we will know whether our predicted ${\hat{y}}_{t}=y_{t}$. In this study, we want to fully utilize all the observed data including the label $y_{t}$ in order to continuously improve the accuracy of the model. Therefore, we designed the novel self-correcting mechanism to further enhance the conventional RNN model. 

### 2.2. Data and Data Preprocessing

We applied our proposed method on the Medical Information Mart for Intensive Care III (MIMIC-III) and Phillips eICU Collaborative Research Dataset. MIMIC-III dataset [17] consists of medical records of over 40,000 ICU patients between 2001 and 2012. Data in MIMIC-III include demographic information, vital signs, medication records, laboratory measurements, observations, fluid balance, procedure codes, diagnostic codes, imaging reports, hospital length of stay, and survival data. We further validated the performance of our proposed method with the Phillips eICU Collaborative Research Dataset. The eICU dataset is populated with data from a combination of multiple ICUs across the United States [18]. The dataset covers 200859 ICU patients admitted in 2014 and 2015. For this project, we extracted the following variables from both the MIMIC-III and eICU datasets:

(1) Demographic information (*static variables*): age and gender
(2) Comorbidities (*static variables*): ICD-9 [19] defined as comorbidity conditions of congestive heart failure, cardiac arrhythmias, valvular disease, pulmonary circulation, peripheral vascular, hypertension, paralysis, neurological disorder, chronic pulmonary diseases, diabetes, hypothyroidism, renal failure, liver diseases, peptic ulcer, aids, lymphoma, metastatic cancer, rheumatoid arthritis, coagulopathy, obesity, fluid electrolyte, anemias, alcohol abuse, drug abuse, psychoses, and depression
(3) Vital signs (*time series variables*): mean arterial blood pressure, heart rate, respiration rate, and temperature
(4) Lab measurements (*time series variables*): bilirubin, BUN (blood urea nitrogen), creatinine, glucose, HCO _3_ (serum bicarbonate), HCT (hematocrit), K (serum potassium), lactate, Mg (serum magnesium), Na (serum sodium), PaCO _2_ (partial pressure of arterial CO _2_), PaO _2_ (partial pressure of arterial O _2_), pH, platelets, troponin, and WBC (white blood cell count)
(5) Fluids (*time series variables*): urine output and fluid balance
(6) Interventions (*time series variables*): usage of mechanical ventilation, vasopressor, and sedative medications

**Figure 1:**
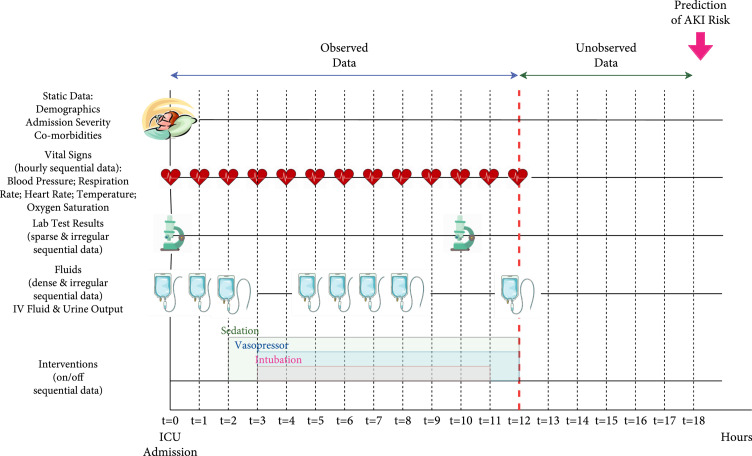
Visualization of data extracted for AKI prediction.

For the extracted time series variables, the vital signs were regularly collected on hourly basis. But the lab measurements, fluid information, and interventions were collected with random time windows. For these variables, we transformed the data into regularly sample time series, where the time gap between two data point is always one hour. For the time steps where there was no recorded data, data were imputed with the weighted average value of the nearest data points. One single data point was obtained on each hour for each patient admitted to ICU. The extracted data are illustrated in Figure 1. We then transformed the data into feature vectors of each patient, normalized the feature vectors with minimal and maximum values in the cohort, and fed them into the models. 

### 2.3. Self-Correcting RNN Models

#### 2.3.1. General Idea

**Figure 2:**
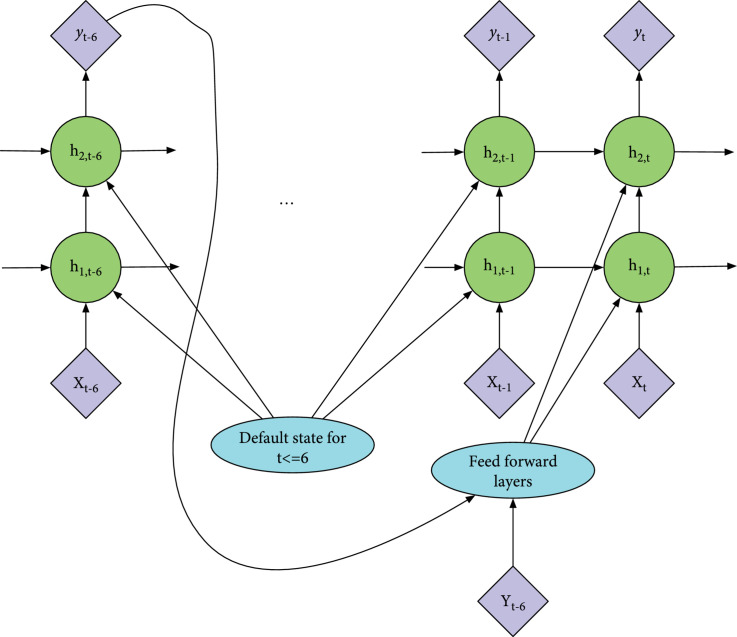
Self-correcting RNN. Circles are used for RNN cells (either LSTM or GRU), while diamond-shaped units are used for input and output. Italic letters (e.g., $x_{t}$ and $y_{t}$) denote the predicted values, while bold capital letters (e.g., $X_{t}$ and $Y_{t}$) denote the actual values.

Figure 2 graphically illustrated the proposed self-correcting RNN framework. Compared to the traditional multilayer RNN, we created a feedback loop between each time step t and t−6,$\forall t\in \left\{7\cdots T\right\}$. At each time step t, we have ${\hat{y}}_{t-6}$, representing the predicted label from our model 6 hours ago, and $y_{t-6}$ the true label. Discrepancies between the predicted ${\hat{y}}_{t-6}$ and the label $y_{t-6}$ are fed into the the feed forward layers. Then, the output of the feed forward layers will be fed into each RNN layer as a part of the input. We believe this can provide additional information about the correctness of previous hidden states.

Note that, for the initial time steps $t\in \left\{1\cdots 6\right\}$, there is no feedback sent to the RNN from the previous time step, as we will need at least 6 hours of data to obtain the true label of AKI. In these cases, a default state is sent to each RNN layer instead. This default state is trained by backpropagation as neural network parameters. Like the normal RNN models, this model can be applied in both classification tasks and regression tasks. Cross-entropy loss and mean square error can be used as the loss functions for classification tasks and regression tasks respectively.

#### 2.3.2. GRU and LSTM Fundamentals

Gated recurrent unit (GRU) and long short-term memory (LSTM) are the two most commonly used variants of RNN. They have been shown to work well on modeling sequential data with long-term dependencies.

The common property shared between GRU and LSTM is the additive update process. The values of the gates depend on the input and the previous state. And update process is controlled by the gates together with the input and previous state. The function $h_{t}=\text{RNN}\left(h_{t-1},x_{t}\right)$ performed by LSTM or GRU can therefore be divided into two steps: $\text{gates}=\text{RN}{\text{N}}_{\text{gate}}\left(h_{t-1},x_{t}\right)$ and $h_{t}=\text{RN}{\text{N}}_{\text{state}}\left(\text{gates},h_{t-1},x_{t}\right)$. The joint distribution of a GRU or LSTM network factorizes as [20,21]

(1)py1:T,h1:T,gate1:T∣x1:T,h0=∏t=1Tpyt∣htpht∣gatet,ht−1,xtpgatet∣ht−1,xt,

where $h_{0}$ denotes the initial states of the GRU/LSTM layers.

Note that these probability distributions modeled by the RNN are all deterministic and that this is the joint distribution of a single-layer RNN. The joint distribution of multilayer RNN factorizes into more components.

#### 2.3.3. Self-Correcting Mechanism

As mentioned in General Idea, at time step t, we will get the true label $y_{t-6}$ (i.e., whether the patient develops AKI). And we want this information to be used to improve the accuracy of the prediction at the current time step. Therefore, the joint probability modeled by the neural network should be

(2)py^1:T,h1:T,gate1:T∣x1:T,h0,y1:T−6=∏t=7Tpy^t∣htpht∣gatet,ht−1,xt,y^t−6,yt−6pgatet∣ht−1,xt,y^t−6,yt−6∏t=16py^t∣htpht∣gatet,ht−1,xtpgatet∣ht−1,xt,

where ${\hat{y}}_{t}$ denotes the output of the neural network and $y_{t}$ denotes the label. $y_{t-6}$ (both the prediction and the actual result) is part of the feature vector we fed into the RNN model at t as the input, together with the original input data. For t<6,$y_{t-6}$ is not available. We replace $y_{t-6}$ with trained constants in that case.

#### 2.3.4. Self-Correcting RNN with Regularization

**Figure 3:**
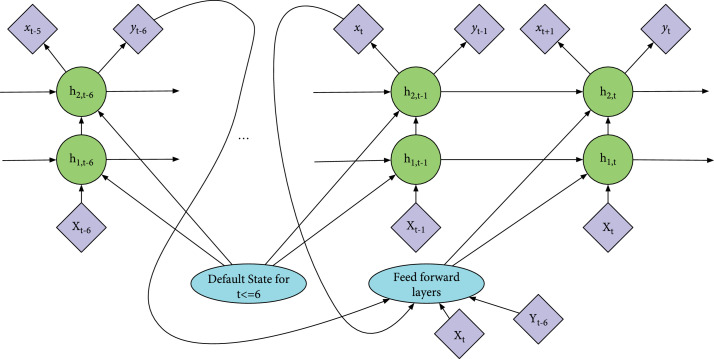
Self-correcting RNN with regularization. Circles are used for RNN cells (either LSTM or GRU), while diamond shaped units are used for input and output. Italic letters (e.g., $x_{t}$ and $y_{t}$) denote the predicted values, while bold capital (e.g., ${\text{X}}_{\text{t}}$ and $Y_{t}$) letters denote the actual values.

Note that for $t\in \left\{1\cdots 6\right\}$, the factorized joint distributions of Equations (1) and (2) are the same. And the difference between Equations (1) and (2) is that, in Equation (2), the probability of $\text{gat}{\text{e}}_{t}$ and $h_{t}$ is conditioned on ${\hat{y}}_{t-6}$ and $y_{t-6}$. Based on this probability model, we designed the neural network shown in Figure 2. Both ${\hat{y}}_{t-6}$ and $y_{t-6}$ are fed into each RNN layer. We call it “self-correcting RNN” because the update of hidden state from $h_{t-1}$ to $h_{t}$ is based on the label $y_{t-6}$ and the predicted value ${\hat{y}}_{t-6}$ from the past time step. If the neural network makes a wrong prediction at the past time step, the hidden state is expected to be updated accordingly. One challenge with the self-correcting RNN is that the label and predicted value used to update $h_{t}$ are from 6 time steps ago. The model may achieve better performance if we can minimize the time gap. To further improve the self-correcting RNN, we designed the regularization method for it. The self-correcting RNN with regularization is shown in Figure 3. Instead of only predicting ${\hat{y}}_{t-1}$ at time step t−1, the model predicts ${\hat{x}}_{t}$ as well. Then, at time step t, the predicted ${\hat{x}}_{t}$ and the input $x_{t}$ will be fed into the feed forward layers together with ${\hat{y}}_{t-6}$ and $y_{t-6}$. The main difference is that the probability distribution of $\text{gat}{\text{e}}_{t}$ and $h_{t}$ now conditions on ${\hat{x}}_{t}$ as well. When we train the model, we add the mean squared error between ${\hat{x}}_{t}$ and $x_{t}$ to the total loss after multiplying it with a certain coefficient. So, the model will learn to predict $x_{t}$ by backpropagation. This regularization method boost the performance of the self-correcting RNN in the following two ways:

(1) It minimizes the time gap of the self-correcting mechanism.
(2) It enforces the model to predict $x_{t+1}$ instead of only $y_{t}$. More information needs to be captured by the hidden state, and hence, the variance of the model decreases.

#### 2.3.5. Self-Correcting Regression RNN with Regularization

All the models described above are classification models because the final result should be a binary value represents whether the patient will develop AKI in the next 6 hours. And the actual AKI label depends on whether the value of urine output/weight is larger than 0.5 mL/kg/h. So, we also designed a self-correcting RNN model with regularization for the urine output regression problem. The structure of this model is the similar to the one shown in Figure 3, except that it predicts the next-6-hour urine output instead of the AKI label and then predicts the label based on the patient’s weight and the predicted urine output. So, it becomes a regression model. In the regression models,$Y_{t}$ is the urine output. And the predicted and actual values of $Y_{t-6}$ are part of the feature vector for t. And the other data (vital signs, lab measurements, etc.) in the feature vector is the same as the classification models. And the mean square error is used for backpropagation.

#### 2.3.6. Stop-Gradient Technique for Feedback Loop

Another challenge with the proposed self-correcting models is that the gradient of $y_{t}$ is affected by the errors at the future time steps. Let $J\left(\theta \right)$ denote the cost function of the parameters θ of the neural network. When we train the RNN models using gradient descent algorithm, the partial derivative $\partial J/\partial {\hat{y}}_{t}$ is first calculated and then backpropagated through the time. In the traditional RNN, the partial derivative of ${\hat{y}}_{t}$ is $\partial J/\partial {\hat{y}}_{t}=\partial J_{t}/\partial {\hat{y}}_{t}$ [22], where $J_{t}$ is the cross-entropy loss between ${\hat{y}}_{t}$ and the label $y_{t}$. The partial derivative of $y_{t}$ is not affected by the loss at the other time steps. This is the desired property of the RNN. In our self-correcting RNN model, the partial derivative of $y_{t}$ is

(3)∂J∂y^t=∂Jt∂y^t+∑i=6T−t−6∂Jt+i∂y^t.

**Figure 4:**
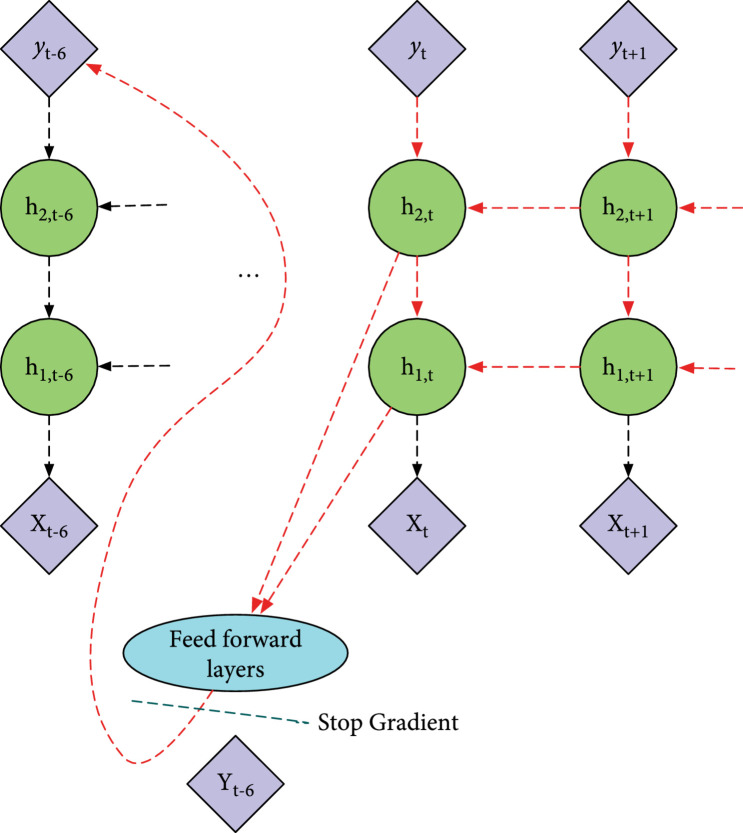
Diagram of backpropagation of the error. Dotted lines indicate the direction of backpropagation, and the dotted lines in red show how the errors in the future time steps are backpropagated to $\hat{y}$ through the feedback loop connection. The green dotted line indicates where we apply the stop-gradient technique.

This is because the loss at the future time step can be backpropagated through the RNN layers and feed forward layers, and finally to $y_{t}$, as shown in Figure 4. And this is not what we desired. Intuitively, the problem is that output layer does not only need to predict ${\hat{y}}_{t}$ accurately but also need to generate the ${\hat{y}}_{t}$ such that the value will later lead to more accurate ${\hat{y}}_{t+6}$ given the current neural network parameters, since $\partial J_{t+6}/\partial {\hat{y}}_{t}$ is also a component of $\partial J/\partial {\hat{y}}_{t}$. This is an undesired property and may potentially affect the performance of the model. The ${\hat{y}}_{t}$ predicted by the model should only be used for the self-correcting mechanism to boost the prediction accuracy at future time step.

To tackle this issue, we truncate the gradient right before feeding the feedback into the feedback network, as shown in Figure 4. This is referred to as the stop-gradient technique for the self-correcting models.

## 3. Results

### 3.1. Experiment Setup

Systematic experiments were conducted to compare the performance of our proposed models and the previously proposed RNN approaches (the baseline models). Two datasets—the MIMIC-III and the eICU datasets—were chosen to validate our proposed methods as they are representative datasets with the richest critical care EHR data. Our models were trained and tested on these two datasets separately.

In this study, we only included patients who stayed in ICU for at least 12 hours. This criterion was set based on two considerations: (1) patients, who were discharged or died within the first 12 hours of ICU stay, were very unique patients that do not fit our clinical application and (2) our proposed self-correcting mechanism only starts from t=7 (the 7th hour in ICU) onward, and thus we will need a number of time steps for the model to be stabilized. In addition, we have also removed patients whose data for the selected variables was not recorded for at least once during the ICU stay. With these inclusion and exclusion criteria, we ended up with about 25,000 patients out of the 40,000 MIMIC patients and about 11,000 patients out of 200,859 eICU patients. We also eliminated the outliers in the extracted time series data (e.g., negative heart rate and unreasonably high body temperature) by removing the extreme data points at the upper or lower one percentiles. We used 5-fold cross-validation to evaluate the performance of all models. 

For each of the two datasets, we trained three variations of our proposed model and also four existing commonly used RNN models as the baseline models. The baseline models included multilayer GRU model, multilayer GRU model with attention mechanism, multilayer LSTM model, and multilayer LSTM model with attention mechanism. We used the self-attention [23] mechanism with same dimension as outputs of each RNN layer. And the three proposed models are self-correcting RNN, self-correcting RNN with regularization, and self-correcting regression RNN with regularization. The proposed self-correcting mechanism added complexity to the RNN. Therefore, to rule out the impacts of added complexity, we built the baseline models with the number of layers from 1 to 3 and the number of neurons in each layer from 64 to 160, shown in Table 1. For each baseline model, we compared the best performance across different architectures with the proposed models built with 2 layers of GRU and 128 neurons in each layer. For all these RNN models, we built the models with the same architecture: two layers of GRU and 128 neurons in each layer. We applied the same settings for dropout, gradient clipping, and reweighted loss function to address the imbalanced dataset. We chose Adam optimization as the optimization method for all the models we trained, as it combines the advantages of Adagrad and RMSProp optimization. We trained all the RNN models with the same initial learning rate and the same decay rate. All the models were trained until they converged. We also adopted dropout method in all the RNN models with the dropout rate of 10% to prevent overfitting. All the seven models converged. 

**Table 1:** Baseline performance with different numbers of layers and neurons.

Model	No. of layers	No. of neurons	MIMIC		eICU	
			AUC	F1	AUC	F1
Multilayer GRU (baseline)	1	64	0.761	0.396	0.739	0.735
		128	0.769	0.415	0.750	0.742
		160	0.773	0.436	0.762	0.746
	2	64	0.769	0.415	0.728	0.730
		128	0.775	0.428	0.753	0.743
		160	0.776	**0.**440	0.757	0.746
	3	64	0.769	0.422	0.723	0.726
		128	0.776	0.434	0.744	0.737
		160	0.779	0.438	0.753	0.741

Multilayer GRU with attention (baseline)	1	64	0.757	0.394	0.761	0.745
		128	0.762	0.422	0.785	0.759
		160	0.766	0.429	0.785	0.758
	2	64	0.760	0.414	0.758	0.745
		128	0.773	0.425	0.793	0.765
		160	0.760	0.409	0.803	0.773
	3	64	0.763	0.413	0.757	0.745
		128	0.768	0.428	0.796	0.768
		160	0.765	0.420	0.814	0.781

Multilayer LSTM (baseline)	1	64	0.733	0.268	0.779	0.754
		128	0.752	0.393	0.788	0.759
		160	0.748	0.384	0.790	0.762
	2	64	0.754	0.403	0.781	0.756
		128	0.762	0.404	0.795	0.766
		160	0.755	0.396	0.803	0.769
	3	64	0.754	0.404	0.780	0.756
		128	0.755	0.358	0.803	0.770
		160	0.758	0.400	0.811	0.777

Multilayer LSTM with attention (baseline)	1	64	0.755	0.383	0.790	0.761
		128	0.760	0.403	0.806	0.773
		160	0.754	0.394	0.811	0.778
	2	64	0.753	0.403	0.808	0.777
		128	0.756	0.398	0.823	0.788
		160	0.761	0.403	0.824	0.788
	3	64	0.747	0.371	0.811	0.779
		128	0.747	0.357	0.824	0.789
		160	0.752	0.398	0.825	0.788

To ensure the reproducibility of our research, our models were tested on two open datasets, MIMIC and eICU, and all the source code for the proposed models will be released online to public as well.

### 3.2. Performance Results

For each dataset, we measured the performance of the models based on area under ROC curve (AUC) and F1 score. We only calculated the AUC for time steps t>6, as our proposed self-correcting mechanism starts at t=7. And we got the result using 5-fold cross-validation. The AUCs were reported in Table 2, and the F1 scores of the models were summarized in Table 3. 

**Table 2:** AUC of the four models on MIMIC-III and eICU.

Model	MIMIC	eICU
Multilayer GRU (baseline)	0.779	0.762
Multilayer GRU with attention (baseline)	0.773	0.814
Multilayer LSTM (baseline)	0.762	0.811
Multilayer LSTM with attention (baseline)	0.760	0.825
Self-correcting RNN ^∗^	0.889	0.837
Self-correcting regression RNN with regularization^∗^	0.886	0.861
Self-correcting RNN with regularization^∗^	0.893	0.871

^∗^Proposed methods.

**Table 3:** F1 scores of the four models on MIMIC-III and eICU.

Model	MIMIC	eICU
Multilayer GRU (baseline)	0.440	0.746
Multilayer GRU with attention (baseline)	0.429	0.781
Multilayer LSTM (baseline)	0.404	0.777
Multilayer LSTM with attention (baseline)	0.403	0.789
Self-correcting RNN^∗^	0.720	0.780
Self-correcting regression RNN with regularization^∗^	0.735	0.803
Self-correcting RNN with regularization^∗^	0.738	0.808

^∗^Proposed methods.

As shown in the tables, all our proposed models outperformed the baseline RNN models over both the MIMIC and eICU datasets. The self-correcting RNN with regularization achieved the best performance. For the MIMIC dataset, our self-correcting RNN with regularization model achieved 0.893 as AUC and 0.738 as F1 score, corresponding to an over 15% improvement comparing with the best baseline models. For the eICU dataset, our self-correcting RNN with regularization model achieved 0.871 as AUC and 0.808 as F1 score, corresponding to close to 10% improvement. 

## 4. Discussions

### 4.1. The Benefits of Self-Correcting Mechanism

Similar RNN methods, such as directed acyclic graph LSTM (DAG-LSTM) and graph state LSTM, have been applied to natural language processing (NLP) tasks [24, 25]. Compared with DAG-LSTM, the proposed self-correcting RNN does not include bidirectional RNN layers that will introduce patient information in the future. In addition, compared with the graph state LSTM that models state transitions for each word, the self-correcting mechanism uses the information from t−6 to t−1 to predict the onset of AKI at time t. 

All the three self-correcting models outperformed the traditional baseline RNN models. From the results on the MIMIC-III dataset, there is a huge difference between the AUC of self-correcting RNN and the traditional multilayer GRU. It is because the additional information provided by the feedback network is helpful for the RNN update process. These results verified our hypothesis that the self-correcting mechanism could boost our model’s performance.

### 4.2. The Benefits of the Proposed Regularization Method

Self-correcting RNN with regularization achieved the highest AUC. And on the eICU dataset, the self-correcting regression RNN with regularization also achieved much higher AUC than the self-correcting RNN model. It indicates that the proposed regularization method helps to further improve the performance of the models by enforcing the model to predict the future input. Our experiment on the eICU dataset verifies that the self-correcting RNN model with regularization has a smaller performance gap between training and testing data, as compared to the one without regularization. It proves that the regularization method reduces the variance of the model.

### 4.3. Why Attention Mechanism Does Not Work

In terms of AUC, the models with attention perform slightly better on eICU than the ones without it but achieved worse results on MIMIC-III. It shows that attention does not help in our case. Attention mechanism is generally helpful in the cases where the output at a time step largely depends on the input at one or a few previous time steps. However, in our case, the output (AKI status) needs to be deduced from the trend of input data and is not just related to the input value at one time step. RNN is able to capture the trend of the data in the hidden states. And our proposed self-correcting mechanism is able to correct the error in the hidden states. So, it works better in our case.

### 4.4. The Benefit of the Stopped Gradient for the Feedback

To verify that the benefits from the proposed stop-gradient technique, we trained a self-correcting RNN with regularization on MIMIC-III without applying the stop-gradient technique. The AUC of this model was 0.852, while the AUC was 0.893 with the stop-gradient technique. The difference in the AUC verified that the stop-gradient technique is critical to the self-correcting models, because it prevent the gradient of $y_{t}$ from being affected by the future errors. 

### 4.5. The Implications of the Proposed Method

The proposed method has the potential to be adopted in clinical applications to prevent AKI and allow intervention to occur in a timely manner. In the clinical settings, the intensivists need to monitor several clinical measurements and prognosis to detect AKI early. The proposed method demonstrated effectiveness in modeling high-dimensional, time series data to alert the clinicians the onset of AKI. The alert is personalized for each patient by modelling the prognosis of multiple clinical variables and the alert time window could be as early as 6 hours, which may enable clinicians to prevent AKI with early treatments. Further studies will be required to validate the effectiveness of the proposed method in prospective trials.

## 5. Conclusions

We proposed a novel self-correcting enhancement to RNNs to better predict the onset of acute conditions in ICU. The proposed self-correcting mechanism made the update process of the hidden state of RNN to be dependent on the previous predicted output and the corresponding label. The additional information provided by the previous predicted output and label helped to boost the performance of model. We also proposed a regularization method for our model, which takes into account not only the model’s prediction errors on the labels but also its estimation errors on the input data. The regularization method reduces the variance of the model and also reduces the time gap for the self-correcting mechanism. The proposed model can be applied on both classification and regression tasks. Our proposed models were tested on real-world large-scale ICU datasets MIMIC-III and eICU and were shown to constantly outperform the baseline multilayer GRU model. Moreover, although we focus on the prediction of acute kidney injury as an example, the proposed model can be easily generalized to predict other acute conditions in ICU. This is the first phase of our project. Inspired by the achieved promising results, we plan to move on further validate the proposed algorithm in our local hospital with the ultimate goal to deploy it as an decision support tool.

## Data Availability

The MIMIC and eICU datasets used in this study are freely available databases for critical care research. The databases could be accessed via https://physionet.org/content/mimiciii/1.4/ and https://eicu-crd.mit.edu/. The source codes for the experiments are publicly available at https://github.com/nus-mornin-lab/AKI_HDS.
